# Comparison of positive pressure ventilation strategies in young children undergoing laparoscopic inguinal hernia repair with laryngeal mask airway: a prospective randomized study

**DOI:** 10.1186/s12871-025-03541-w

**Published:** 2025-12-19

**Authors:** Mariana AbdElSayed Mansour, Hatem ElMoutaz Mahmoud, Hebatallah NegmEldeen AbdElAzeem, Dina Mahmoud Fakhry

**Affiliations:** Department of Anesthesiology, Surgical Intensive Care and Pain Management, Faculty of Medicine, Beni-Suef University, Beni-Suef, Egypt

**Keywords:** Laparoscopic, Inguinal hernia, Laryngeal mask, PCV, VCV, PCV-VG

## Abstract

**Background:**

A laryngeal mask is a viable alternative to tracheal intubation for airway control in pediatric day surgery. Therefore, the methodology for respiratory management using a laryngeal mask in mechanically ventilated patients is of particular significance. Here, we compare pressure-controlled ventilation (PCV), volume-controlled ventilation (VCV), and pressure-controlled ventilation with volume guaranteed (PCV-VG) in terms of respiratory mechanics in children who underwent laparoscopic inguinal hernia repair at various time intervals.

**Methods:**

This prospective, randomized, comparative study was conducted on 90 children aged 1–5 years who underwent elective laparoscopic inguinal hernia repair and had an American Society of Anesthesiologists (ASA) physical status of I or II. They were assigned to the PCV, VCV, or PCV-VG groups (30 patients per group). The primary outcome measure was peak inspiratory pressure (PIP). Secondary outcome measures were other parameters of respiratory dynamics, hemodynamic parameters and adverse respiratory events in the postoperative period.

**Results:**

The PIP was significantly lower in the PCV and PCV-VG groups compared to the VCV group at all measured time points. For example, at T1, PIP was lower in the PCV group (11.2 ± 0.8 cmH_2_O) and the PCV-VG group (11.7 ± 0.9 cmH_2_O) compared to the VCV group (13.7 ± 2.1 cmH_2_O) (*p* < 0.001). Similarly, also at T3, it was lower in the PCV (15.5 ± 1.5 cmH_2_O) and the PCV-VG group (15.8 ± 1.3 cmH_2_O) compared to the VCV group (17.6 ± 2.4 cmH_2_O) (*p* < 0.001). Additionally, plateau pressure (Pplat) and respiratory airway resistance (Raw) were significantly lower, while dynamic compliance (Cdyn) was significantly higher in the PCV and PCV-VG groups compared to the VCV group at all time points. At T2, Pplat was notably lower in the PCV group (10.6 ± 0.8 cmH_2_O) and the PCV-VG group (13.5 ± 1.4 cmH_2_O) compared to the VCV group (15.3 ± 2.5 cmH_2_O) (*p* < 0.001). Cdyn was significantly higher in the PCV group and the PCV-VG group (e.g., at T2, PCV: 19.9 ± 2.7 mL/cmH_2_O; PCV-VG: 20.8 ± 3.5 mL/cmH_2_O) compared to the VCV group (15.3 ± 2.6 mL/cmH_2_O; *p* < 0.001). No significant differences were observed among the groups regarding expired tidal volume (VTe), end-tidal CO_2_ (EtCO_2_), dead space ratio (Vd/Vt), hemodynamics, or postoperative respiratory adverse effects.

**Conclusion:**

In pediatric patients undergoing laparoscopic surgeries, PCV and PCV-VG are superior to VCV as evidenced by lower PIP and higher dynamic compliance (Cdyn).

**Trial registration:**

This trial was registered prospectively on ClinicalTrials.gov (NCT06612125) on September 25, 2024.

## Introduction

Inguinal hernia repair is one of the most common surgical procedures performed on pediatric patients [1, 2]. Laparoscopic hernia repair was introduced following technological advancements that enabled pediatric surgeons to adopt less invasive techniques [3]. Pneumoperitoneum with CO_2_ can affect the cardiopulmonary system in multiple ways, including a reduction in lung volumes, an increase in mean arterial pressure (MAP), and CO_2_ absorption, which may lead to acidosis. These physiological changes can result in various forms of cardiac distress [4, 5].

Maintaining a secure airway is paramount in pediatric laparoscopic surgery. Traditionally, orotracheal intubation (OTI) has been the standard for airway management in these procedures. However, the use of a laryngeal mask airway (LMA) has emerged as a potential alternative. Studies investigating the efficacy and safety of LMA compared to OTI in pediatric laparoscopic inguinal surgery have shown promising results [6]. Specifically, research indicates that LMA does not lead to statistically significant differences in blood gas test results or airway pressures when compared to OTI. Furthermore, recovery times are notably faster with LMA, suggesting an advantage in patient extubation and post-operative care. These findings collectively support the use of LMA as a safe and effective tool for airway maintenance in pediatric laparoscopic surgery [7]. A recent meta-analysis, in particular, provides strong evidence for the safety and effectiveness of LMA in pediatric laparoscopic inguinal hernia repair, demonstrating no greater safety risks than endotracheal tube (ETT) and showing shortened anesthesia and recovery times [8]. The use of a laryngeal mask as an alternative to tracheal intubation for airway control in outpatient surgery has been well established. Therefore, proficiency in respiratory management with a laryngeal mask is essential. Additionally, mechanical ventilation is frequently employed for airway management in clinical settings. However, the superiority of one ventilation strategy over another remains unclear [9].

The fundamental modes of mechanical ventilation are volume-controlled ventilation (VCV) and pressure-controlled ventilation (PCV) [10]. VCV ensures a target ventilation volume through continuous flow but may increase peak inspiratory pressure (PIP) during gas insufflation [11]. In PCV mode, the ventilator maintains a steady pressure by reducing the flow rate, and the ventilation volume fluctuates according to the patient's respiratory mechanics [12]. pressure-controlled ventilation with volume guaranteed (PCV-VG) combines the benefits of VCV and PCV, ensuring a consistent ventilation volume through a decelerating flow pattern [13].

Here, we compared VCV, PCV, and PCV-VG in young children who underwent laparoscopic inguinal hernia repair with LMA. The primary outcome measure was peak inspiratory pressure (PIP), while secondary outcome measures were other parameters of respiratory dynamics, hemodynamic parameters and adverse respiratory events in the postoperative period.

## Methods

This prospective randomized trial was conducted at Beni-Suef University Hospital from October 2024 to March 2025. It received approval from the Department of Anesthesiology, Surgical Intensive Care, and Pain Management, as well as the ethics committee (both at the Faculty of Medicine, Beni-Suef University, FM-BSU; REC/01092024/Mansour). It was registered on ClinicalTrials.gov (NCT06612125) on September 25, 2024. Ninety eligible patients, aged 1–5 years with an American Society of Anesthesiologists (ASA) physical status of I-II, were scheduled for laparoscopic inguinal hernia surgery under general anesthesia. This study adhered to the Helsinki Declaration, and written informed consent was obtained from their legal guardians.

The exclusion criteria included: (1) cardiopulmonary disease; (2) severe hepatorenal dysfunction; (3) an upper respiratory tract infection within two weeks before the procedure; (4) overweight status (exceeding 20% of standard body weight); (5) neuromuscular disease; (6) an anticipated difficult airway; and (7) hiatus hernia or gastroesophageal reflux disease.

Patients were randomly assigned to PCV, VCV, or PCV-VG ventilation modes (30 per group) using a computer-generated random number sequence prepared by a statistician. Upon enrollment, cases were systematically recorded on a random number table to determine their group assignment. A randomization table was created using Random Allocation software (v1.0.0) by M. Saghaei from the Department of Anesthesia, Isfahan University of Medical Sciences (Isfahan, Iran). Since the study's design was open-label, there was no blinding. However, this did not introduce assessment bias because all data points were objective, numerical readouts directly from a calibrated anesthesia machine monitor, not subjective interpretations.

### Anesthetic technique

A comprehensive preoperative clinical evaluation was conducted the day before the procedure. All patients fasted according to the institutional regimen during the recruitment phase: six hours for light meals and four hours for breast milk. No premedication was administered. Standard monitoring devices were applied upon entry to the operating room, including noninvasive blood pressure measurement, electrocardiography, capnography, pulse oximetry, and end-tidal gas analysis. Anesthesia was induced via inhalation of 8% sevoflurane with a fresh gas flow of 8 L/min of oxygen, followed by intravenous (IV) line placement. An appropriately sized LMA was inserted after confirming unresponsiveness to the jaw thrust [14] using the index finger method, and the cuff was inflated. Correct LMA positioning was verified by symmetrical chest excursion during bag-mask ventilation, an appropriately squared-off EtCO_2_ tracing, bilateral chest auscultation, the absence of an audible leak at 20 cmH_2_O, and the absence of perceptible abdominal distension. The LMA was secured with tape over the maxilla. Anesthesia was maintained with sevoflurane at 1.5–2% in 100% oxygen, along with fentanyl (1 µg/kg) and rocuronium bromide (0.6 mg/kg). Patients were then allocated to the PCV, VCV, or PCV-VG group (30 patients per group). Radial artery was cannulated for arterial blood gas samples.

In the VCV group (*n* = 30), patients underwent VCV with a target tidal volume (VT) of 7 mL/kg, a respiratory rate of 16 breaths/min, and an inspiratory-to-expiratory (I:E) ratio of 1:2.

In the PCV group (*n* = 30), patients received PCV, with PIP adjusted to achieve a VT of 7 mL/kg, an I:E ratio of 1:2, and a respiratory rate of 16 breaths/min, with a maximum PIP of 20 cmH_2_O.

Patients in the PCV-VG group (*n* = 30) were subjected to pressure-controlled ventilation with volume guaranteed (PCV-VG), targeting a VT of 7 mL/kg, an I:E ratio of 1:2, and a respiratory rate of 16 breaths/min.

No positive end-expiratory pressure (PEEP) was applied throughout the procedure in any group. A maximum PIP of 20 cmH_2_O was maintained across all groups. After the procedure, IV paracetamol (6–15 mg/kg) was administered. Neuromuscular blockade was reversed using atropine (20 µg/kg) and neostigmine (50 µg/kg) [We did not use acceleromyography or electromyography to objectively assess the reversal of neuromuscular block due to limitations in equipment availability]. Once adequate ventilation was achieved, the LMA was removed.

During the recovery period, patients were monitored for the occurrence of adverse events, including breath-holding, bronchospasm, laryngospasm, coughing, and desaturation. Patients with a Modified Aldrete score exceeding nine were transferred from the post-anesthesia care unit to the clinical ward.

The following data were recorded:

Patient characteristics: Age, gender, weight, and ASA physical status.

Procedure durations:

- Anesthesia duration: From induction to patient recovery.
- Surgery duration: From skin incision to final suture.
- Insufflation duration: From initiation to conclusion of intra-abdominal CO_2_ insufflation.
- Recovery time: From cessation of sevoflurane inhalation to discharge from the operating room to the post-anesthesia care unit.

#### Primary outcome measure

Peak Inspiratory Pressure (PIP). *Secondary outcome measures*: Other parameters of respiratory dynamics including Plateau pressure (Pplat), dynamic compliance (Cdyn), respiratory airway resistance (Raw), expired tidal volume (VTe), EtCO_2_, and the Vd/Vt ratio (estimated using Bohr’s formula: Vd/Vt = (PaCO_2_ – PEtCO_2_)/PaCO_2_). All data, including Cdyn were obtained directly from the anesthesia machine's integrated monitor._._ Additionally, Mean Arterial Pressure (MAP), heart rate (HR), and postoperative respiratory complications (such as sore throat, hypoxemia, cough, hoarseness, bronchospasm, and laryngospasm) have been evaluated.

Data collection intervals:

- T1**:** Post-anesthesia induction and before CO_2_ pneumoperitoneum initiation.
- T2**:** 5 min after pneumoperitoneum initiation.
- T3**:** 30 min after pneumoperitoneum initiation.
- T4**:** At the conclusion of pneumoperitoneum.

### Sample size

The sample size was calculated by comparing PIP among different PPV strategies (VCV, PCV, and PC-VG) in young children undergoing laparoscopic inguinal hernia repair with LMA. As reported in a previous study [15], the mean ± SD of PIP in the VCV group was 14.83 ± 2.92 cmH_2_O, while in the PCV group, it was 12.66 ± 2.09 cmH_2_O, and in the PC-VG group, it was 12.56 ± 2.06 cmH_2_O. The minimum required sample size was determined to be 26 children per group to reject the null hypothesis with 80% power at an α level of 0.05, using a one-way analysis of variance (ANOVA) test. Sample size calculation was performed with G*Power software (version 3.1.9.6) for MS Windows, developed by Franz Faul at Kiel University, Germany. To account for potential attrition, the study included 30 subjects per group.

### Statistical methods

Normally distributed numerical data were presented as mean ± SD, while qualitative (categorical) data were expressed as frequencies (number of cases) and percentages. The Kolmogorov–Smirnov test was used to assess the normality of numerical data. The ANOVA test was applied to compare numerical variables between study groups as all continuous variables were normally distributed. The chi-square (χ^2^) test will be employed to compare categorical data, with the exact test applied when expected frequencies are below 5. A p-value < 0.05 was considered statistically significant. All statistical analyses were performed using Microsoft Excel 2019 (Microsoft Corporation, NY, USA) and IBM SPSS (Statistical Package for the Social Sciences; IBM Corp, Armonk, NY, USA) version 22 for Microsoft Windows.

## Results

**Fig. 1:**
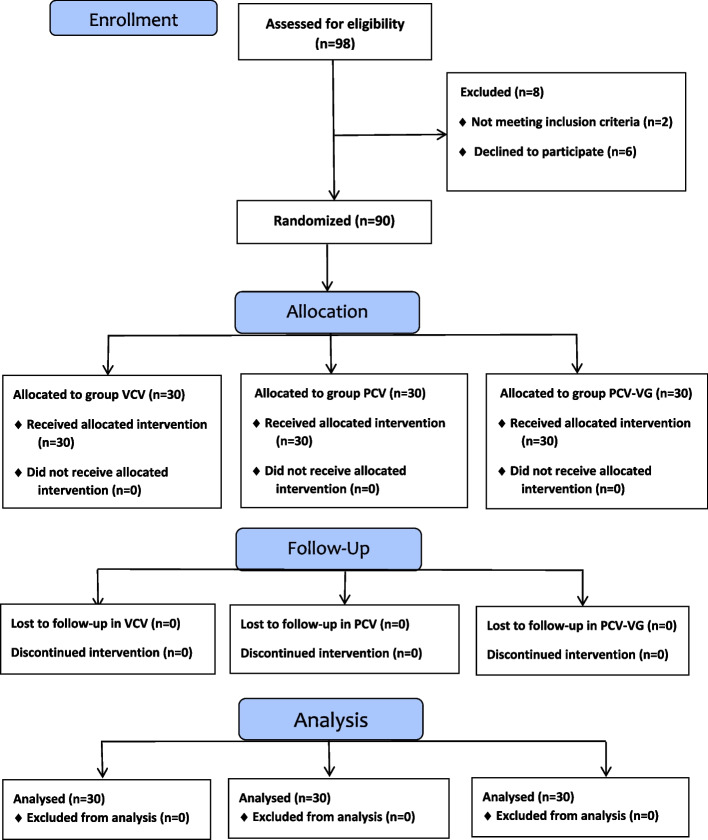
CONSORT flow diagram

A total of 98 individuals were assessed for eligibility. Of these, 96 met the inclusion criteria. The parents of 90 individuals provided informed consent to participate, while the parents of the remaining 6 eligible individuals did not. These 90 consenting participants were then randomly allocated into three groups of thirty, as illustrated in the CONSORT flow diagram (Fig. 1). All study participants completed the trial. Table 1 presents demographic data and operative characteristics. No significant differences were observed among the three groups regarding mean age, body mass index (BMI), or ASA physical status. Surgical duration and recovery time were compared across the groups. The PCV group exhibited a significantly prolonged anesthesia duration compared to the other groups (*p* < 0.001). Additionally, insufflation duration was significantly shorter in the PCV group compared to the PCV-VG group (*p* = 0.044).

**Table 1:** Demographic data

	**VCV** **(** ***n*** ** = 30)**	**PCV** **(** ***n*** ** = 30)**	**PCV-VG** **(** ***n*** ** = 30)**	***p*** **-value**
Age (months)	30.2 ± 11.5	29.9 ± 11.7	29.9 ± 11.2	0.994
Gender:				
Females	2 (6.7%)	1 (3.3%)	1 (3.3%)	0.770
Males	28 (93.3%)	29 (96.7%)	29 (96.7%)	
Weight (kg)	13.7 ± 3.0	13.5 ± 2.3	13.2 ± 2.4	0.776
ASA I	25 (83.3%)	27 (90.0%)	25 (83.3%)	0.698
ASA II	5 (16.7%)	3 (10.0%)	5 (16.7%)	
Anesthesia duration (minutes)	57.5 ± 3.4	53.0 ± 5.0^a^	58.0 ± 6.4	< 0.001
Operative duration (minutes)	48.2 ± 4.2	46.2 ± 5.3	48.1 ± 6.7	0.271
Insufflation duration (minutes)	41.5 ± 3.8	39.0 ± 5.5^b^	42.3 ± 6.0	0.044
Recovery time (minutes)	5.4 ± 1.2	5.3 ± 1.6	5.4 ± 1.1	0.956

Data are presented as the mean ± standard deviation (SD) or the number of patients (%). A *p*-value < 0.05 is considered significant, and a *p*-value > 0.05 is considered nonsignificant ^a^significant difference to the other groups and ^b^significant difference to the PCV-VG group

*Abbreviations*: *VCV* Volume Controlled Ventilation, *PCV* Pressure Controlled Ventilation, *PCV-VG* Pressure Controlled Ventilation-Volume Guaranteed, *ASA* American Society of Anesthesiologists

**Fig. 2:**
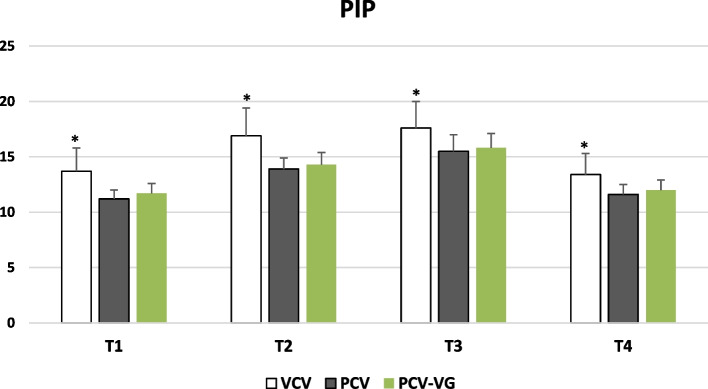
Mean peak inspiratory pressure (PIP) in the 3 study groups over the study period (*Statistically significant difference compared to the other 2 groups)

Table 2 displays PIP, Pplat, Cdyn, Raw, VTe, EtCO_2_, and Vd/Vt across the three ventilation modes (VCV, PCV, and PCV-VG) at time points T1, T2, T3, and T4. PIP was significantly lower in PCV and PCV-VG modes than in VCV (*p* < 0.001); however, no significant difference was noted between PCV and PCV-VG at any time point (*p* > 0.05) (Fig. 2). Additionally, Pplat was significantly lower in PCV and PCV-VG than in VCV at T1 and T2 (*p* < 0.001). However at T3 and T4, only the PCV group exhibited significantly lower Pplat compared to VCV (*p* < 0.05).

**Table 2:** Respiratory mechanics

	**VCV** **(** ***n*** ** = 30)**	**PCV** **(** ***n*** ** = 30)**	**PCV-VG** **(** ***n*** ** = 30)**	***p*** **-value**
PIP-T1	13.7 ± 2.1^a^	11.2 ± 0.8	11.7 ± 0.9	< 0.001
PIP-T2	16.9 ± 2.5^a^	13.9 ± 1.0	14.3 ± 1.1	< 0.001
PIP-T3	17.6 ± 2.4^a^	15.5 ± 1.5	15.8 ± 1.3	< 0.001
PIP-T4	13.4 ± 1.9^a^	11.6 ± 0.9	12.0 ± 0.9	< 0.001
Pplat-T1	12.1 ± 2.1^a^	10.4 ± 0.7	10.5 ± 2.0	< 0.001
Pplat-T2	15.3 ± 2.5^a^	10.6 ± 0.8	13.5 ± 1.4	< 0.001
Pplat-T3	15.7 ± 2.5^b^	14.0 ± 1.5	15.0 ± 1.4	0.003
Pplat-T4	11.8 ± 1.7^b^	10.9 ± 0.8	11.1 ± 1.0	0.031
Cdyn-T1	17.7 ± 2.3^a^	22.2 ± 2.5	22.9 ± 3.6	< 0.001
Cdyn-T2	15.3 ± 2.6^a^	19.9 ± 2.7	20.8 ± 3.5	< 0.001
Cdyn-T3	14.8 ± 2.8^a^	19.2 ± 2.4	20.1 ± 3.6	< 0.001
Cdyn-T4	18.0 ± 2.6^a^	22.2 ± 2.1	23.0 ± 2.9	< 0.001
Raw-T1	23.3 ± 3.1^a^	17.5 ± 3.1	17.7 ± 2.8	< 0.001
Raw-T2	28.4 ± 2.3^a^	19.0 ± 3.6	19.2 ± 3.0	< 0.001
Raw-T3	28.6 ± 2.3^a^	19.6 ± 3.7	19.8 ± 3.2	< 0.001
Raw-T4	23.3 ± 3.0^a^	18.0 ± 3.5	18.2 ± 3.1	< 0.001
VTe-T1	100.3 ± 8.8	100.0 ± 12.2	100.4 ± 14.5	0.991
VTe-T2	102.8 ± 12.6	97.4 ± 15.2	94.5 ± 15.3	0.082
VTe-T3	101.3 ± 12.9	96.9 ± 14.1	93.7 ± 15.4	0.119
VTe-T4	106.6 ± 11.5^c^	100.6 ± 13.5	95.7 ± 14.7	0.008
EtCO_2_-T1	33.4 ± 2.4	33.5 ± 2.1	34.0 ± 1.9	0.479
EtCO_2_-T2	38.1 ± 2.6	37.8 ± 2.4	37.4 ± 2.1	0.484
EtCO_2_-T3	38.8 ± 2.1	38.3 ± 2.1	37.7 ± 2.0	0.100
EtCO_2_-T4	33.5 ± 2.2	33.3 ± 1.8	33.8 ± 1.3	0.468
Vd/Vt-T1	0.118 ± 0.037	0.127 ± 0.048	0.129 ± 0.044	0.569
Vd/Vt-T2	0.125 ± 0.043	0.119 ± 0.053	0.122 ± 0.044	0.883
Vd/Vt-T3	0.118 ± 0.045	0.115 ± 0.041	0.121 ± 0.043	0.864
Vd/Vt-T4	0.119 ± 0.043	0.127 ± 0.043	0.131 ± 0.044	0.559

Data are presented as the mean ± standard deviation (SD). *P*-values < 0.05 were considered significant and *p*-values > 0.05 were considered nonsignificant. ^a^significant difference to the other groups; ^b^significant difference to PCV group; and ^c^significant difference to PCV-VG

*Abbreviations*: *VCV* Volume-Controlled Ventilation, *PCV* Pressure-Controlled Ventilation, *PCV-VG* Pressure-Controlled Ventilation-Volume Guaranteed, *PIP* Peak Inspiratory Pressure (cmH_2_O), *Pplat* Plateau Pressure (cmH_2_O), *Cdyn* Dynamic Compliance (ml/ cmH_2_O), *Raw* Airway Resistance (cmH_2_O/L/s), *VTe* Expired Tidal Volume (ml), *EtCO2* End-tidal Carbon Dioxide (mmHg)

**Fig. 3:**
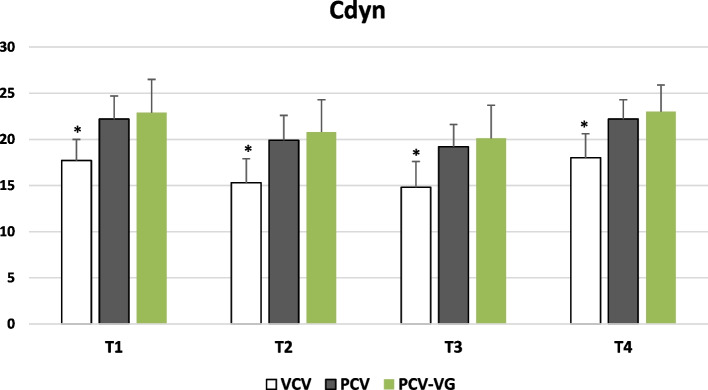
Mean dynamic compliance (Cdyn) in the 3 study groups over the study period (*Statistically significant difference compared to the other 2 groups)

Cdyn was significantly higher in PCV and PCV-VG modes than in VCV at all time points (*p* < 0.001), with no significant difference between PCV and PCV-VG (Fig. 3). Raw was significantly lower in PCV and PCV-VG than in VCV at all time points (*p* < 0.001), with no significant difference between PCV and PCV-VG. No significant difference in VTe was found among the three ventilation modes at T1, T2, and T3 (*p* > 0.05); however, at T4, VCV exhibited a significantly higher VTe than PCV-VG (*p* < 0.05). No significant differences were observed between EtCO_2_ and Vd/Vt groups at any time point (*p* > 0.05).

Table 3 presents hemodynamic parameters, including MAP, HR, and SpO_2_, which showed no significant differences among the three groups at any time point (*p* > 0.05). Additionally, respiratory adverse events did not differ significantly between ventilation modes (*p* > 0.05) (Table 4).

**Table 3:** MAP, HR, and SpO_2_

	**VCV** **(*n* = 30)**	**PCV** **(*n* = 30)**	**PCV-VG** **(*n* = 30)**	***p*-value**
MAP-T1	80.6 ± 3.7	81.3 ± 5.6	83.1 ± 6.0	0.150
MAP-T2	75.1 ± 2.3	76.0 ± 4.0	76.2 ± 3.3	0.383
MAP-T3	73.5 ± 2.0	74.1 ± 4.6	74.6 ± 4.5	0.531
MAP-T4	81.1 ± 2.3	82.4 ± 4.6	81.7 ± 4.7	0.475
HR-T1	104 ± 8	106 ± 6	104 ± 4	0.475
HR-T2	97 ± 5	99 ± 6	99 ± 6	0.436
HR-T3	93 ± 5	95 ± 7	95 ± 6	0.169
HR-T4	99 ± 5	101 ± 6	100 ± 5	0.312
SpO_2_-T1	98.7% ± 0.5%	98.8% ± 0.4%	98.7% ± 0.4%	0.466
SpO_2_-T2	98.5% ± 0.6%	98.6% ± 0.6%	98.7% ± 0.5%	0.410
SpO_2_-T3	98.5% ± 0.6%	98.6% ± 0.6%	98.7% ± 0.5%	0.419
SpO_2_-T4	98.8% ± 0.4%	98.9% ± 0.3%	98.7% ± 0.5%	0.474

Data are presented as the mean ± standard deviation (SD). A p-value < 0.05 is considered significant, and a *p*-value > 0.05 is considered nonsignificant

*Abbreviations*: *MAP* Mean Arterial Pressure, *HR* Heart Rate, *SpO2* Oxygen Saturation, *VCV* Volume Controlled Ventilation, *PCV* Pressure Controlled Ventilation, *PCV-VG* Pressure Controlled Ventilation - Volume Guaranteed

**Table 4:** Respiratory adverse events

	VCV (*n* = 30)	PCV (*n* = 30)	PCV-VG (*n* = 30)	*p*-value
Cough	3 (10.0%)	3 (10.0%)	1 (3.3%)	0.538
Hoarseness	3 (10.0%)	1 (3.3%)	0 (0.0%)	0.160
Laryngospasm	3 (10.0%)	1 (3.3%)	0 (0.0%)	0.160
Bronchospasm	0 (0.0%)	0 (0.0%)	0 (0.0%)	N/A
Hypoxemia	2 (6.7%)	0 (0.0%)	0 (0.0%)	0.129

Data are presented as the number of patients (%). A *p*-value < 0.05 is considered significant, and a *p*-value > 0.05 is considered nonsignificant

## Discussion

Previous studies have compared VCV and PCV-VG ventilation in adult patients [16]; however, the effects of respiratory dynamics in pediatric patients receiving LMA ventilation with VCV and PCV-VG remain unclear. During pneumoperitoneum, intrathoracic pressure increases, leading to atelectasis in dependent lung regions, early airway closure, increased PIP, reduced lung volumes, and decreased Cdyn [17]. Clinical studies suggest that PCV may provide a higher VT at a lower PIP during laparoscopic procedures, particularly when PIP is elevated in VCV mode [18]. PCV is recommended for reducing airway and lung pressures in cases with a high risk of elevated PIP, such as neonates and patients with emphysema [19, 20].

This study aimed to compare VCV, PCV, and PCV-VG in young children undergoing laparoscopic inguinal hernia repair with LMA. Measurements were taken at four time points: after anesthesia induction and before the initiation of CO_2_ pneumoperitoneum (T1), five minutes after pneumoperitoneum initiation (T2), 30 min after pneumoperitoneum initiation (T3), and after pneumoperitoneum deflation (T4). The primary outcome measures was PIP respiratory dynamics parameters, while secondary outcome measures included other respiratory dynamics parameters, hemodynamic parameters and postoperative respiratory complications.

Our findings demonstrated that PCV and PCV-VG outperform VCV in terms of lower PIP, Pplat, and Raw, as well as higher Cdyn. No significant differences in VTe, EtCO_2_, or Vd/Vt were observed among the studied groups. Similarly, the three ventilation modes showed no differences in hemodynamic parameters or postoperative respiratory complications.

Consistent with our results, Ikeidan et al. reported that PCV results in lower peak inspiratory airway pressures while maintaining ventilation comparable to VCV in pediatric patients undergoing LMA ventilation. No significant differences were observed in hemodynamic parameters between the two groups at all time points. The PCV-VG group exhibited a significant reduction in both PIP and Pplat compared to the VCV group. Pulmonary Cdyn was significantly higher in the PCV-VG group than in the VCV group. No significant differences in respiratory complications were noted between the VCV and PCV groups [21]. Similarly, Huan Liu et al. [22] studied 64 pediatric patients undergoing laparoscopic surgery with LMA ventilation. They found that PCV-VG demonstrated superior performance over VCV at an equivalent ventilation ratio, with a lower PIP and a slightly reduced mean pressure (Pmean), without significant differences in oxygenation levels. These results align with previous studies comparing PCV, VCV, and PCV-VG, which showed that PCV-VG reduces PIP and Pmean while maintaining similar oxygenation levels [23, 24]. Moreover, PCV-VG allows effective oxygenation, precise VT control, and improved PIP regulation [25]. The consistency across these pediatric studies, including our own, likely stems from the inherent advantages of pressure-controlled modes in delivering a set tidal volume within a controlled pressure limit. This is particularly beneficial in pediatric patients, who have smaller, more compliant airways and are more susceptible to barotrauma from high pressures. The ability of PCV-VG to guarantee a tidal volume while adapting pressure breaths to lung mechanics appears to be a key factor in these consistent positive outcomes. Further supporting these advantages, Kothari et al. [26] found PCV-VG to be associated with superior lung Cdyn and lower PIP than VCV during laparoscopic cholecystectomy, with lung ultrasonography also indicating better lung aeration [27].

Maroun Badwi et al. [15] similarly found that PCV-VG was superior to VCV in reducing peak inspiratory pressure (PIP) and improving dynamic lung compliance (Cdyn) in non-paralyzed patients with laryngeal mask airways (LMAs) under anesthesia. However, Badwi et al. also noted no significant differences between PCV and PCV-VG when anesthesia depth was adequate in patients with normal pulmonary function (ASA I or II). The findings of Lee et al. [28] complement our own by demonstrating that PCV-VG resulted in lower PIP and improved Cdyn compared to VCV, suggesting that it may serve as an effective alternative mechanical ventilation mode for patients in the prone position undergoing lumbar spine surgery. Our study adds to this body of evidence by demonstrating similar physiological responses across different surgical settings. This broad consistency suggests a fundamental advantage of PCV-VG mode in optimizing ventilation parameters and potentially reducing the risk of ventilator-induced lung injury in various surgical scenarios.

The present study had some limitations. First, infants younger than one year old were excluded. This age limit was deliberately chosen to minimize the inherent physiological challenges associated with anesthesia and surgery in very young infants and neonates, who exhibit unique respiratory mechanics that can significantly confound the assessment of ventilation strategies. Consequently, our findings may not be directly generalizable to this vulnerable population. Second, no extrinsic PEEP was applied in the ventilation strategies, necessitating an end-expiratory hold to measure auto-PEEP intraoperatively [29]. This potentially limiting the clinical applicability of our findings in practices that routinely use PEEP. Additionally, patients with pre-existing respiratory conditions were not included. This exclusion was necessary to ensure a homogeneous study population and minimize confounding variables related to underlying lung pathology, but it limits the generalizability of our results to the broader pediatric surgical population. Future studies should address these limitations, and further research is needed to clarify the impact of different ventilation strategies on respiratory mechanics across diverse patient populations.

## Conclusion

PCV and PCV-VG ventilation modes are preferred over VCV in pediatric patients undergoing laparoscopic procedures due to their lower PIP, Pplat, and airway resistance, as well as their superior lung Cdyn. However, no significant differences were observed in VTe, ETCO_2_, or Vd/Vt among the three ventilation modes. Additionally, hemodynamic parameters and respiratory adverse effects did not differ significantly between the studied groups.

## Data Availability

The data are available upon reasonable request from the corresponding author.

## Abbreviations

PCV: Pressure-controlled Ventilation
VCV: Volume-controlled Ventilation
PCV-VG: Pressure-controlled Ventilation-Volume Guaranteed
ASA: American Society of Anesthesiologists
Pplat: Plateau Pressure
PIP: Peak Inspiratory Pressure
Raw: Respiratory Airway Resistance
VTe: Expired Tidal Volume
EtCO_2_: End Tidal CO_2_
Vd/Vt: Dead Space Ratio
MAP: Mean Arterial Pressure
LMA: Laryngeal Mask Airway
PPV: Positive Pressure Ventilation
IV: Intravenous
PEEP: Positive End-Expiratory Pressure
Cdyn: Dynamic Compliance
HR: Heart Rate
SD: Standard Deviation
BMI: Body Mass Index
SPO_2_: O_2_ Saturation
Pmean: Mean airway pressure
OTI: Orotracheal intubation
ETT: Endotracheal tube
